# Perivascular macrophages in high-fat diet-induced hypothalamic inflammation

**DOI:** 10.1186/s12974-022-02519-6

**Published:** 2022-06-09

**Authors:** Natalia F. Mendes, Licio A. Velloso

**Affiliations:** 1 Laboratory of Cell Signaling, Obesity and Comorbidities Research Center, University of Campinas, Rua Carl Von Linnaeus s/n, Instituto de Biologia - Bloco Z. Campus Universitário Zeferino Vaz - Barão Geraldo, Campinas, SP 13083-864 Brazil; 2 National Institute of Science and Technology on Neuroimmunomodulation, Rio de Janeiro, Brazil

**Keywords:** Hypothalamus, Obesity, Monocytes, Microglia, Inflammation

## Abstract

Brain macrophages and microglia are centrally involved in immune surveillance of the central nervous system. Upon inflammatory stimuli, they become reactive and release key molecules to prevent further damage to the neuronal network. In the hypothalamic area, perivascular macrophages (PVMs) are the first line of host defence against pathogenic organisms, particles and/or substances from the blood. They are distributed throughout the circumventricular organ median eminence, wrapping endothelial cells from fenestrated portal capillaries and in the hypothalamic vascular network, where they are localised in the perivascular space of the blood–brain barrier (BBB). Some studies have indicated that PVMs from the hypothalamus increase the expression of inducible nitric oxide synthase and vascular endothelial growth factor upon feeding for a long time on a high-fat diet. This adaptive response contributes to the impairment of glucose uptake, facilitates BBB leakage and leads to increased lipid and inflammatory cell influx towards the hypothalamic parenchyma. Despite these early findings, there is still a lack of studies exploring the mechanisms by which PVMs contribute to the development of obesity-related hypothalamic dysfunction, particularly at the early stages when there is chemotaxis of peripheral myeloid cells into the mediobasal hypothalamus. Here, we reviewed the studies involving the ontogeny, hallmarks and main features of brain PVMs in vascular homeostasis, inflammation and neuroendocrine control. This review provides a framework for understanding the potential involvement of PVMs in diet-induced hypothalamic inflammation.

## Background

Hypothalamic inflammation plays a key role in the development of diet-induced obesity (DIO) and subsequent systemic metabolic abnormalities [1, 2]. Studies have shown that hypothalamic microglia are implicated in the initial phase of this process [2, 3]. As early as a few hours after the introduction of a high-fat diet (HFD), free fatty acids (FFAs) rise in the arcuate nucleus of the hypothalamus (ARC) as a consequence of increased transport through the fenestrated capillaries at the median eminence (ME); this process results in the activation of ARC microglia [2–4]. In this context, microglia undergo rapid transcriptional and morphological changes which lead to the activation of a multi-layered inflammatory response, promoting increased chemokine, cytokine, reactive oxygen species (ROS) and nitric oxide (NO) in the hypothalamic microenvironment [5–7].

If the consumption of a fat-rich diet persists for several weeks, there is the recruitment of peripheral monocyte-derived macrophages, such as CD169 + and CCR2 +, from the blood and cerebrospinal fluid (CSF) into the hypothalamic parenchyma [5, 7]. This cellular migration is triggered by chemokines, including fractalkine (CX3CL1) and monocyte chemoattractant protein-1 (MCP-1, also known as CCL2), in response to the increased levels of FFAs and inflammatory cytokines in the neural tissue [8, 9].

At the onset of the inflammatory response, other immune cells, such as neutrophils, lymphocytes and natural killer T cells, can reach the hypothalamic parenchyma in a time-dependent manner [10, 11]. HFD-induced BBB leakage facilitates the entrance of peripheral cells into the CNS [12]. The mechanisms underlying BBB disruption in DIO are complex and depend on several factors, such as the type and duration of injury and altered function/structure of the neurovascular unit (NVU) [13]. Thus, BBB integrity depends on a strict architecture, comprehending tight junctions on the endothelial cells of the blood capillaries, a perivascular space separating the basal membrane of these capillaries where both pericytes and PVMs reside, and the terminal feet of astrocytes [14, 15].

Due to their anatomical location, PVMs are directly involved in immune surveillance and, consequently, in controlling the passage of substances and immune cells through the BBB [16]. In a recent study, Lee et al. [5] observed an increased inducible nitric oxide synthase (iNOS) production by PVMs and parenchymal lysozyme M (LysM)-expressing myeloid cells in the ARC/ME unit from long-term HFD-fed mice, resulting in BBB leakage and higher vascular permeability, thus facilitating FFA accumulation in the hypothalamic parenchyma.

Despite recent advances in the characterisation of mechanisms underlying peripheral cell infiltration in the diet-induced hypothalamic inflammation, it is still unknown how PVMs are involved in these processes, especially in the initial phase of the inflammatory response. Here, we briefly reviewed the studies that have defined the ontogeny, hallmarks and functions of these cells to provide a framework for understanding their potential involvement in diet-induced hypothalamic inflammation.

## Main text

### Classification and ontogeny of resident immune cells of the CNS

The resident immune cells of the CNS, also known as brain-resident macrophages, can be defined as microglia and non-parenchymal brain macrophages [17, 18]. Microglial cells are widely distributed throughout the brain parenchyma, whereas non-parenchymal brain macrophages are the cells that reside in the areas surrounding barrier or border regions, such as the meninges, perivascular space and choroid plexus (ChP) stroma [18–20]. Due to their anatomical distribution, non-parenchymal macrophages are also known as central nervous system-associated (CAMs) or border-associated macrophages (BAMs). Unlike peripheral macrophages that infiltrate the CNS, microglia and non-parenchymal cells reside in the brain under homeostatic conditions, controlling tissue homeostasis and innate immune defence. Together, they make up the first line of host defence against cellular or pathogenic components.

The development of brain-resident macrophages involves haematopoiesis in two major sites; the embryonic yolk sac and foetal liver [18]. At embryonic day 9.5 (E9.5), microglia derive from primitive haematopoietic cells present in the yolk sac and populate the neuroepithelium [21]. Under homeostatic conditions, microglia undergo renewal through a low rate of proliferation in combination with apoptosis [22]. On the other hand, non-parenchymal macrophages (perivascular, meningeal, or ChP macrophages) originate from yolk sac, foetal liver-derived progenitor cells and bone marrow at E10.5 for the brain parenchyma and E11.5 for the ChP [23–26].

PVMs and meningeal macrophages sustain their population by a minimal turnover [25]. Although ChP is replenished by haematopoietic stem cells (HSCs), recent fate-mapping studies have revealed steady monocyte trafficking to the ChP throughout adult life to renew resident macrophages [16]. At E13.5, the BBB is established, blocking the entrance of the foetal liver monocytes into the brain parenchyma, at least under homeostatic conditions [25].

### PVMs regulation of the hypothalamic microvascular network

**Fig. 1:**
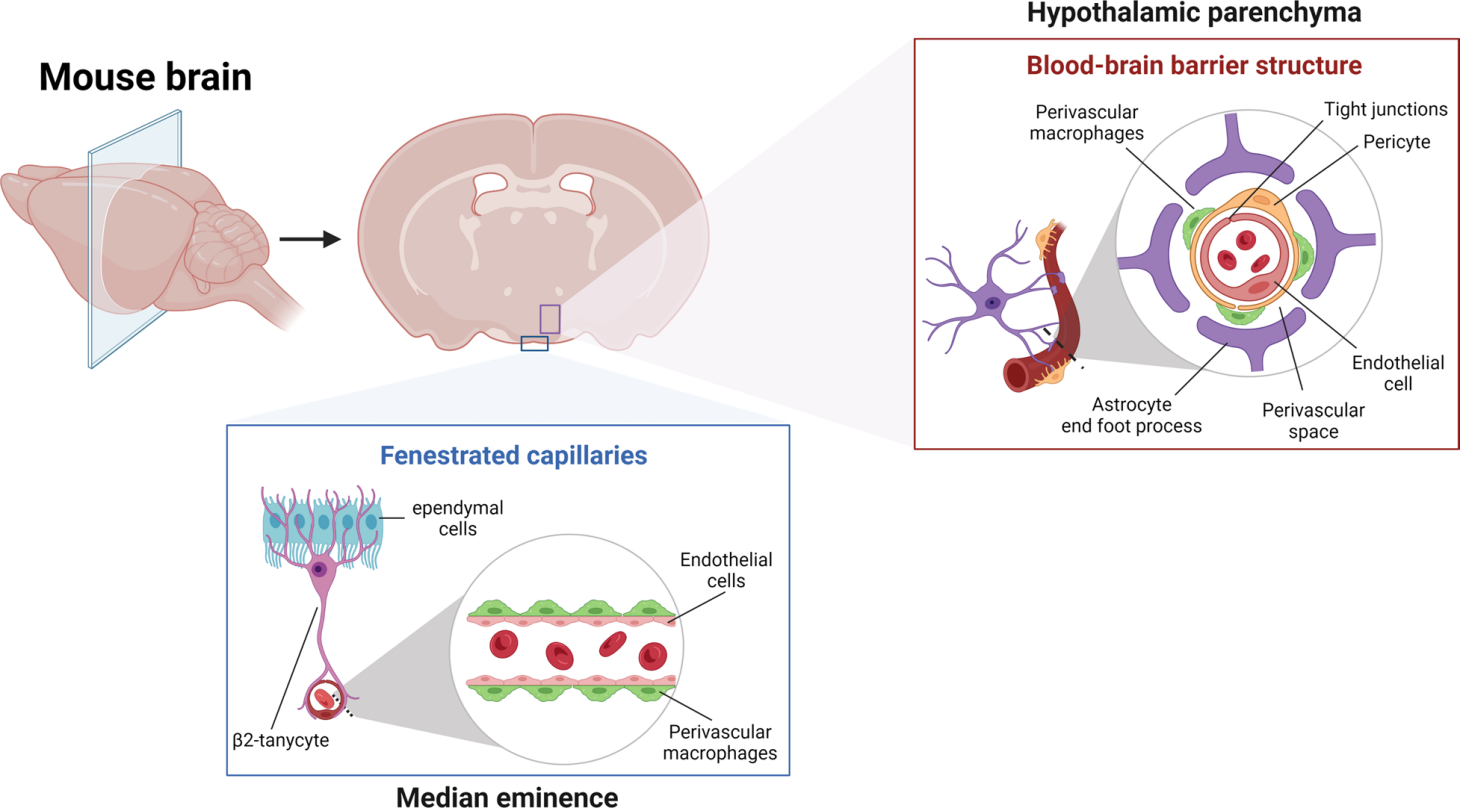
PVMs distribution in the hypothalamic vascular network. In the median eminence, PVMs are widely distributed wrapping endothelial cells from the fenestrated capillaries. Throughout the hypothalamic parenchyma, they belong to the neurovascular unit of the BBB. BBB integrity and function depends (1) on the presence of tight junctions between the cerebral endothelial cells, which form a barrier, selectively excluding blood-borne substances from entering the brain parenchyma, and (2) of PVMs wrapping the endothelial cells in the perivascular space, which are essential for immune surveillance. BBB architecture also comprehends astrocyte end-feet enclosing the capillary, and pericytes embedded in the capillary basement membrane

Brain PVMs are located within the perivascular space surrounding arterioles and venules and the Virchow-Robin space (VRS), a CSF-filled perivascular compartment [27, 28]. The perivascular space presents distinct functions: it works as a drainage system for substances and waste products from the CSF and also as a barrier limiting the entrance of peripheral cells into the parenchyma [29, 30]. Among the brain-circulation barrier regions, PVMs interact with diverse cell types, such as astrocytes, pericytes and endothelial cells (Fig. 1).

PVMs are able to extend their processes along with the perivascular space. Studies have reported their involvement in various inflammatory conditions, such as experimental autoimmune encephalomyelitis (EAE) [31], cerebral malaria [32] and diet-induced inflammation [5, 33], which indicates the ability of PVMs to directly sense molecular cues in the blood and regulate vascular permeability.

The contribution of PVMs to vascular homeostasis has been investigated in recent years [34–36]. Even though the mechanisms behind the PVM control of vascular permeability specifically in the ME are not fully described, at least in other circumventricular organs (CVOs) devoid of a BBB, such as the area postrema, PVMs are known to restrict the entry of tracers larger than 10 kDa [37]. As for the brain PVMs, mesenteric PVMs and pericytes are abundantly distributed and wrap around the endothelial cells of small blood vessels and capillaries. The depletion of PVMs from the mesenteric vessels increases vascular permeability [35]. Additionally, in the mouse retina, PVMs can move along the vessels, aggregating blood-borne molecules and keeping the structure of the blood-retina barrier intact [38].

PVMs from the hypothalamic area impact vascular permeability depending on nutritional and hormonal stimuli. HFD-fed mice show increased blood vessel density and length in the hypothalamus, which is indicative of angiopathy [39]. Hypothalamic angiogenesis is mediated by the hypoxia-inducible factor-1α (HIF-1)/vascular endothelial growth factor (VEGF) pathway, activated by the action of leptin on astrocytes, which has physical contact with PVMs [40]. Pericytes play a crucial role by mediating leptin entrance into the hypothalamic parenchyma [41]. Although pericytes also make contact with PVMs, their crosstalk in HFD-induced vascular network disruption was not investigated yet.

Hypothalamic myeloid cells and PVMs have been also described as important sources of VEGF [33], contributing to the increased microvessel permeability and tight junction complex reorganization in the ARC/ME unit, and facilitating the access of metabolic substrates to the hypothalamic neuronal network [42]. PVMs from the ARC/ME unit also increase iNOS and decrease endothelial NOS (eNOS) production with a long-term HFD, resulting in increased vascular permeability and BBB leakage [5].

PVMs can also regulate the access of hormones and nutrients to hypothalamic neurons. According to Ciofi et al. [43], there are at least three ways by which hormones and nutrients can access the ARC. The first involves transcytosis from the plasma to brain parenchyma through endothelial and glial components of the BBB. The second occurs via the glymphatic system, also known as the paravascular pathway, by which a variety of molecules exit the porous capillaries of the neighbouring ME and dissipate within the cerebrospinal fluid, bathing the coalescent perivascular spaces of the ARC/ME region. The third is found throughout the subependymal plexus (SEP), a specific vascular route irrigating the ARC for rapid exchange and encompassing capillary afferents to the ARC anastomosed with the intra-infundibular capillary loops of the ME.

In diet-induced hypothalamic inflammation, these processes can be affected, at least in part due to angiopathy. The reasons why and whether PVMs facilitate angiopathy development and BBB disruption upon a HFD, and how changes in surrounding cells, such as astrocytes and pericytes, affects PVMs upon inflammatory stimuli are not fully elucidated. However, it is known that both processes start at the onset of the HFD-induced inflammatory response, which supports the hypothesis that PVMs from the ARC/ME are important components of the complex system that drive the early stages of hypothalamic inflammatory response and not just the late response. Thus, the involvement of PVMs in the vascular network disruption in the initial phase of the hypothalamic inflammation requires further investigation.

### PVMs involvement in hypothalamic inflammation and peripheral myeloid cell chemotaxis

Meningeal and ChP macrophages share several functions with PVMs [44]. However, PVMs are most likely involved in the HFD-induced hypothalamic inflammation and peripheral cell infiltration due to their characteristic anatomical distribution wrapping the fenestrated capillaries from ME and in the perivascular spaces from BBB of the parenchymal hypothalamic vascular network [5, 45].

Some mechanisms can contribute to the increased content of proinflammatory cytokines in the brain parenchyma, such as the entrance across the BBB, stimulation of different CVO, nerve stimulation and the release from infiltrating or resident immune cells [46]. The ME is a CVO that is richly irrigated by fenestrated capillaries that secure the permeability to the blood/spinal fluid interface (BSFI) [47]. Hence, along with microglia, PVMs sense minimal changes in peripheral signals, such as hormone levels (e.g., leptin and insulin) and nutrients in the blood, quickly triggering an inflammatory response and morphological/functional changes. In addition, PVMs express receptors involved in cytokine responsiveness, phagocytosis and antigen presentation, primarily coordinating innate and adaptive immune responses upon inflammatory signals within the CNS [48, 49].

Cytokines, enzymes and growth factors have their expression increased by PVMs from multiple tissues in response to inflammatory stimuli and injury conditions [50–53]. In the CNS of rodents, a long-term HFD intake (20 weeks) increases the hypothalamic mRNA expression of hypoxia-inducible factor-1α (HIF-1α) and proinflammatory cytokines IL-1β, IL-6 and TNF-α, while decreasing the expression of vascular endothelial growth factor-A (VEGF-a) [5]. Likewise, Jais et al. [33] observed the transient expression of VEGF by PVMs in the hypothalamus of HFD-fed mice, which was increased three days after the introduction of a HFD and reduced after a chronic period of HFD (6 months).

These short-lived changes in the expression of cytokines and other molecules during distinct phases of inflammatory responses are not restricted to PVMs. HFD-induced hypothalamic inflammatory processes also occur in a transient biphasic manner. Following up to one week of a HFD, the levels of proinflammatory cytokines and chemokines increase in the hypothalamic parenchyma [2, 4, 9, 54]. Upon maintenance on the HFD for two weeks or longer, the levels of inflammatory markers decrease and are re-established at higher levels only after four weeks of a HFD [2, 3, 6]. These variations in the expression of pro-inflammatory signals by PVMs and other cells in the hypothalamus are due to the intensity of the stimuli and mainly to the phase of the inflammatory response, which specifically implicate on changes on oxygen availability, angiogenesis, phagocytosis, cell proliferation and activation, tissue remodelling, and other inflammatory-related process.

PVMs also facilitate the communication between injured CNS parenchyma and circulating immune cells and molecules [55]. Due to their phagocytic activity, PVMs display intracellular lipid droplets in HFD-fed conditions, preventing excessive lipid accumulation in the hypothalamic extracellular space [5], which is detrimental to neurons and other cells. Accordingly, when lipid clearance mediated by microglia is insufficient to prevent FFA accumulation in the parenchyma, PVMs from the perivascular spaces, with higher phagocytic activity, are recruited to the ARC parenchyma and acquire a phenotype of microglia-like cells, which is known as parenchymal myeloid cells (LysM + cells). These microglia-shaped parenchymal PVMs express the macrophage marker CD169. Thus, linear-shaped CD169 + PVMs undergo a phenotypic switch to microglia-like cells just after migrating from the perivascular space to the hypothalamic parenchyma. The molecular mechanisms by which PVMs initiate this phenotypic switch and how they move or are attracted into the hypothalamic parenchyma remain to be studied.

### PVM involvement in hypothalamic neuroendocrine control

#### Central regulation of glucose metabolism

PVMs contribute considerably to glucose homeostasis. Jais et al. [33] observed that mice fed a HFD present a reduction in expression of the glucose transporter 1 (GLUT1) in hypothalamic vascular endothelial cells, resulting in reduced glucose uptake into the CNS. The authors show that this suppression of endothelial GLUT1 is transient and restored upon prolonged consumption of a HFD, which depends on the compensatory VEGF production by myeloid cells, including PVMs. The selective ablation of VEGF in myeloid cells, by crossing lysozyme-Cre (LysM-Cre) driver mice with Vegf-flox mice, reduces endothelial GLUT1 and brain glucose uptake.

In the study conducted by Lee et al. [3], the inhibition of hypothalamic iNOS, released mainly by PVMs, not only reduced inflammatory markers and LysM + infiltrating cells in the HFD-fed mice, but also improved glucose intolerance and systemic insulin resistance in obese mice. These changes in insulin resistance are aligned with metabolic phenotypes of Nos2 knockout mice (Nos2^−/−^), in which the gene encoding iNOS is disrupted [56]. Thus, based on these findings it is reasonable to assume that hypothalamic PVMs have distinct actions in metabolic regulation in response to the consumption of a HFD.

#### Hypothalamic–pituitary–adrenal (HPA) axis regulation

PVMs have an important role in the regulation of HPA adaptation upon systemic inflammatory stimuli [57]. Circulating cytokines can boost the abluminal expression of distinct soluble mediators, such as prostaglandin E2 (PGE2), an important mediator of the inflammatory response, by binding to the luminal surface of endothelial cells associated with small venules along the surface of the brain [58, 59]. After the systemic administration of IL-1β or lipopolysaccharide (LPS), for example, there is increased PGE2 and cyclooxygenase-2 (COX-2) synthesis in cerebral endothelial cells, which depends on the activation of PVMs [60, 61].

Depletion of PVMs, on the other hand, leads to a reduced expression of COX-2 and PGE2 by endothelial cells, reinforcing the importance of their crosstalk in HPA axis regulation [48]. Moreover, the increased expression of PGE2 by PVMs in the hypothalamus increases sympathetic nervous system activity in the paraventricular nucleus (PVN), increasing blood pressure [53, 62].

**Fig. 2:**
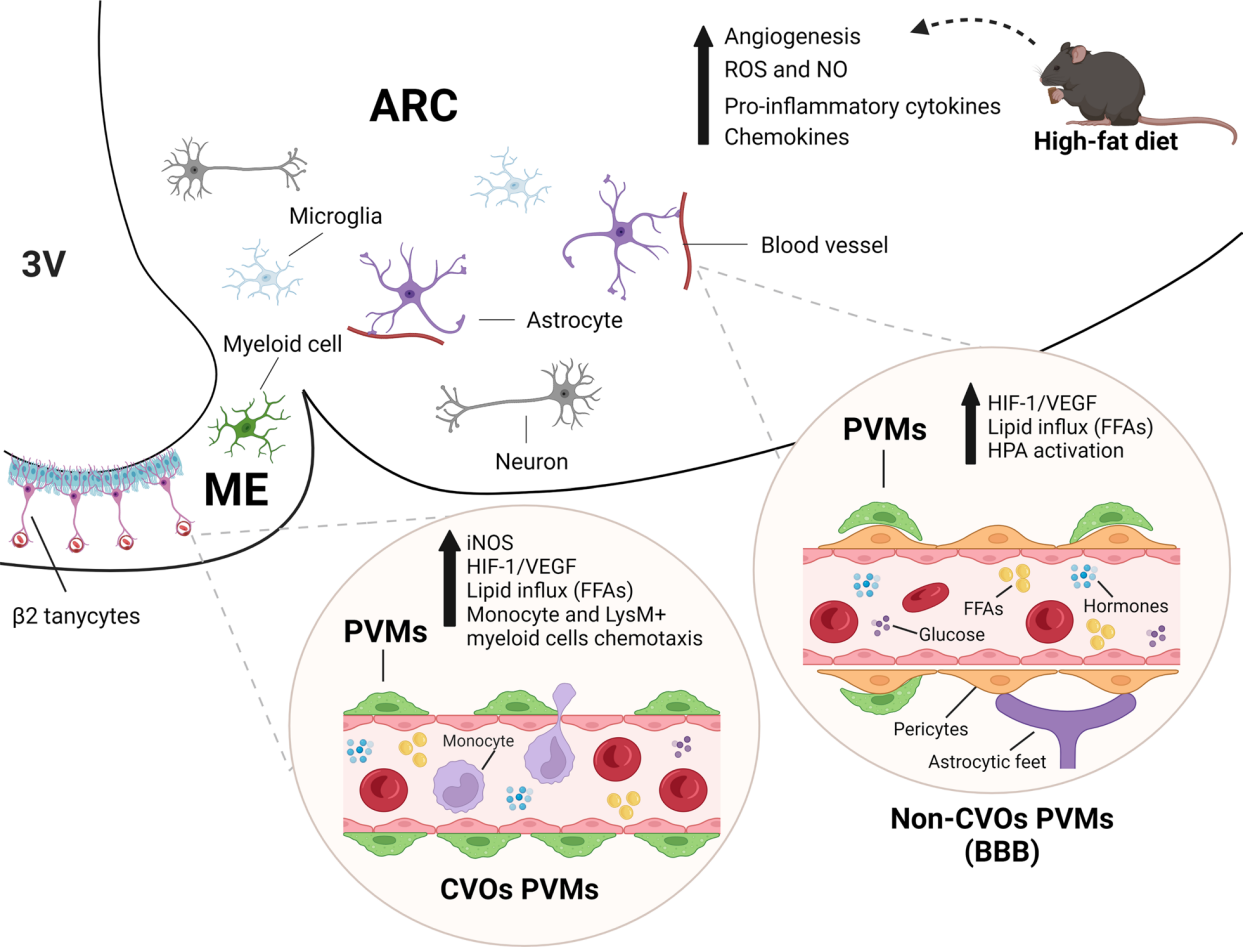
PVMs are involved in the increased iNOS and VEGF synthesis, lipid influx, and changes in glucose uptake in the hypothalamus of mice fed a high-fat diet. The hypothalamic inflammatory response under a high-fat feeding comprehends several processes, which depend on glial and non-glial cells interactions, and results in increased expression of pro-inflammatory cytokines, chemokines, ROS and NO production and angiogenesis. PVMs are distributed in the perivascular spaces from BBB throughout the hypothalamic parenchyma and wrapping fenestrated capillaries in the ME. Their anatomical distribution allows them to play a critical role in immune defence. PVMs increase the expression of iNOS and VEGF upon a long-term feeding on a HFD, resulting in increased free fatty acids (FFAs) influx into the hypothalamic parenchyma. HPA activation is observed in mice after inflammatory stimuli, such as the ones caused by LPS and IL1-β injection. This neuroendocrine modulation depends on PVMs and endothelial cells, however whether a HFD can result in the same adaptive response remains unexplored

**Fig. 3:**
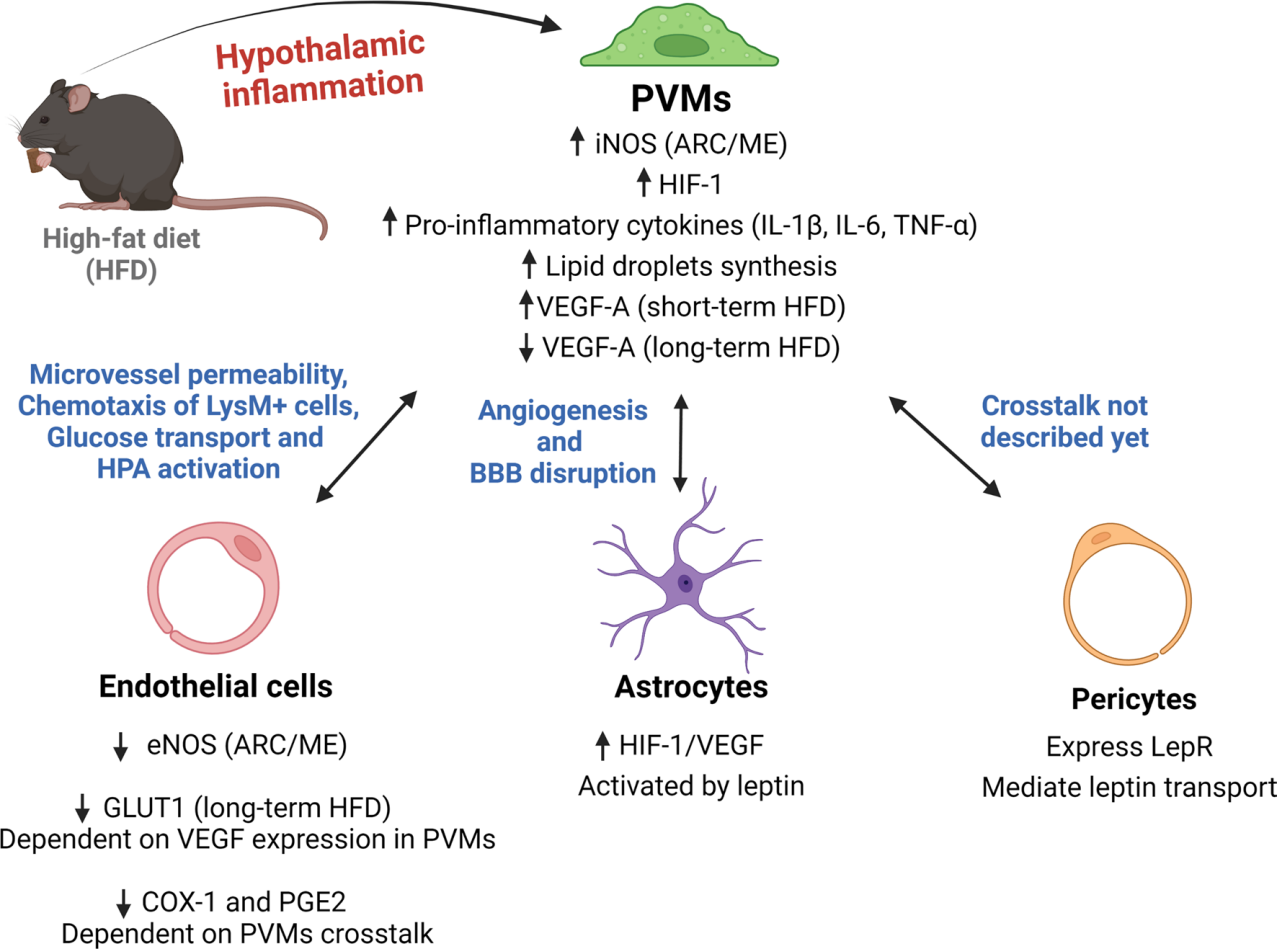
PVMs molecular adaptations in response to a high-fat diet, and their crosstalk with surrounding cells. The hypothalamic inflammatory response triggered by the consumption of a high-fat diet for a short or long time promotes changes in PVMs and in the cells that make contact with them. The crosstalk between PVMs and endothelial cells regulates microvessel permeability, throughout iNOS and eNOS production, facilitating LysM + myeloid cells infiltration into the hypothalamic parenchyma; reducing glucose transport through reduction of GLUT1, and changing the HPA axis throughout modulation of COX-1 and PGE2 expression on endothelial cells. The astrocytes, on the other hand, mediate angiogenesis throughout the activation of HIF1-VEGF signaling under leptin action. Pericytes express leptin receptor (LepR) and play a critical role by mediating leptin transport into the hypothalamus, but their crosstalk with PVMs is still unexplored

PVMs and endothelial cells may have some opposite actions, which are observed in the regulation of prostaglandins and IL-6 expression upon systemic LPS challenge [63]. The expression of IL-1 receptor type 1 (IL-1R1) on endothelial cells, but not PVMs, for example, is important to induce HPA activation upon systemic IL-1β stimulation [57]. Still, whether a locally-restricted PVM batch in the hypothalamus modulates the HPA axis and how they play their roles remain to be elucidated. The involvement of PVMs in HFD-induced hypothalamic inflammation and neuroendocrine functions is shown in Fig. 2, while their detailed adaptations and crosstalk with surrounding cells during HFD-induced hypothalamic inflammation is shown in Fig. 3.

### Brain PVMs transcriptomic signature

Transcriptome profiling has been widely applied to determine the particularities hidden behind thousands or even millions of cells that compose given tissues and organs. Despite microglia display a transcriptional signature that distinguishes them from other myeloid populations in both humans and mice [64–66], only a few studies have separately examined the transcriptomic profile of brain PVMs.

Zeisel et al. [67] pioneered these analyses by evaluating the brains of rodents. Employing a single-cell RNA sequencing approach (scRNA-seq), they revealed that both microglia and PVMs express the brain macrophage markers *Aif1* and *Cx3cr1*, whereas PVMs are distinguished from microglia via the expression of *Mrc1* and *Lyve1*. In addition, the genes *Lyl1* and *Spic* were found to be specific to PVMs when compared with other CAMs.

To explore the transcriptional program and dynamics of the macrophages in the CNS, Goldmann et al. [16] also performed a scRNA-seq of microglia, PVMs, their precursors, monocytes and peritoneal macrophages. They found that PVMs and microglia were transcriptionally closely related, whereas monocytes and peritoneal macrophages had a distinct RNA profile. Briefly, they observed that all myeloid populations express *Cx3cr1*, *Csf1r* and *Aif1* genes, whereas PVMs are distinguishable from microglia based on their expression of *Mrc1* and *Cd36*. Moreover, microglia expressed higher levels of *P2ry12* and *Hexb* and lower levels of *Ptprc* (encoding CD45) than PVMs.

When comparing the transcriptomic signatures of microglia with engrafted parenchymal brain macrophages from bone marrow (BM) or haematopoietic stem cell (HSC) transplantation, Shemer et al. [66] observed that the transcriptomic profile of graft-derived cells shows considerable overlap with the transcriptome of PVMs, including changes in the gene expression of *ApoE*, *Msn4a7*, *Slc2a5* and *Sall1*, respectively.

Using the same single-cell sequencing approach, Jordão et al. [68] unravelled the complexity of the CNS myeloid landscape and the dynamics of several myeloid populations during neuroinflammation (EAE experimental mouse model). They performed a scRNA-seq from CD45 + cells isolated from distinct CNS compartments (leptomeninges, perivascular space and parenchyma and choroid plexus) and observed that, in the peak of the EAE, PVMs show some similarities with reactive microglia. They display the reduced gene expression of *Lyve1*, *Cbr2*, *Folr2*, *Ccl8*, *Ctsd*, *Cd163*, *F13a1* and *H2-Eb1* when compared to the naive state. On the other hand, *Cd74*, *Ccl5*, *Ciita*, *H2-Aa*, *H2-Ab1* and *Plbd1* were shown to be highly expressed by PVMs in the peak of the EAE. Their data suggest that homeostatic subsets of CNS macrophages, including PVMs, can quickly change their phenotypes and generate context- and time-dependent subsets, which is similar to that observed in microglial cells [69, 70]. Likewise, PVMs transcriptomic signature presented by the studies previously mentioned [16, 66–68] may vary depending on distinct characteristics, such as; the brain area analysed, the interactions of PVMs with surrounding cells, the sex and age of the mice or subjects, and the health/disease condition. Thus, the data interpretation always should consider these aspects.

To date, only one study has unveiled the transcriptome profile of different cell types from the ARC/ME upon chow diet and HFD [71]. Unfortunately, in their analysis, microglia and PVMs were not clustered independently. Thus, it is still unclear which disease-specific PVMs subsets exist and what their transcriptional profiles and dynamics upon HFD-induced inflammation are. Future studies should investigate their specific transcriptomic signature in more detail, using sorting-based approaches to isolate PVMs from other CAMs and microglia in the hypothalamic tissue.

Some state-of-art approaches have been developed and will allow this detailed analysis in the future. Kim et al. [72] have recently generated two transgenic mice harbouring the split Cre fragments: Cx3cr1^ccre^:Sall1^ncre^ mice, which target microglia and Cx3cr1^ccre^:Lyve1^ncre^ mice, which target a subset of PVMs. After a single peripheral LPS challenge, they performed mRNA sequencing from brain cell lysates. The differential gene expression analysis revealed several genes that were distinctly modulated in both cell populations. Thus, similar approaches can help researchers interested in isolating microglia from PVMs in the hypothalamus to more deeply investigate the features of these cell populations in diet-induced inflammation or even in other inflammatory conditions. Table 1 shows the main markers of brain PVMs identified in rodents.

**Table 1:** PVMs hallmarks identified in the brain of rodents

Genes	Cells and brain regions	References
*Lyl1, Spic, Mrc1 and Lyve1*	Whole tissue from primary somatosensory cortex (S1) and hippocampal CA1 region	Zeisel et al. 2015 [67]
*Mrc1* and *Cd36*	Microglia and PVMs from the brain cortex (without meninges and ChP)	Goldmann et al. 2016 [16]
*ApoE, Ms4a7, Slc2a5, and Sall1*	Microglia and bone marrow-derived parenchymal CNS macrophages of whole brain from bone marrow chimeras	Shemer et al. 2018 [66]
Downregulation of *Lyve1*, *Cbr2*, *Folr2*, *Ccl8*, *Ctsd*, *Cd163*, *F13a1* and *H2-Eb1; and u*pregulation *of Cd74, Ccl5, Ciita, H2-Aa, H2-Ab1* and *Plbd1*	CD45 + cells isolated from leptomeninges, perivascular space and parenchyma, and ChP from an EAE mouse model	Jordão et al. 2019 [68]

## Conclusions

PVMs have undisputed roles in the regulation of hypothalamic inflammation in response to a HFD; however, despite the persistent worldwide increase in the prevalence of obesity, there have been few studies exploring the details of PVMs involvement in this process. PVMs are strategically distributed in the perivascular spaces where they play a critical role in immune-defence and in other functions such as, mediating vascular homeostasis and neuroendocrine regulation. These peculiarities place them alongside other glial cells in the centre of the responses of the hypothalamus to the consumption of a HFD. Understanding the specific roles of these cells in the hypothalamic homeostasis and their involvement in diet-induced hypothalamic inflammation may provide advances in the understanding of hypothalamic pathology in obesity as well as in the development of new strategies to deal with the harmful effects of a HFD. Future studies aimed at defining the transcriptomic signature and detailed functional features of PVMs may provide advances in this field.

## Data Availability

Not applicable.

## Abbreviations

AIF1: Allograft inflammatory factor 1
APOE: Apolipoprotein E
ARC: Arcuate nucleus of the hypothalamus
BAMs: Border-associated macrophages
BBB: Blood–brain barrier
CAMs: CNS-associated macrophages
CBR2: Carbonyl reductase
CCL2: C–C motif chemokine 2
CCL5: C–C motif chemokine 5
CCL8: C–C motif chemokine 8
CCR2: C–C chemokine receptor type 2
CD36: Cluster of differentiation 36
CD74: Cluster of differentiation 74
CD163: Cluster of differentiation 163
CD169: Cluster of differentiation 169
ChP: Choroid plexus
CIITA: Class II major histocompatibility complex transactivator
CNS: Central nervous system
CSF1R: Colony stimulating factor 1 receptor
CTSD: Cathepsin D
CVO: Circumventricular organ
CX3CR1: CX3C chemokine receptor 1
DIO: Diet-induced obesity
EAE: Experimental autoimmune encephalomyelitis
F13A1: Coagulation factor XIII a chain
FOLR2: Folate receptor beta
GLUT1: Glucose transporter 1
H2-AA: Histocompatibility 2, class II antigen A, alpha
H2-AB1: Histocompatibility 2, class II antigen A, beta 1
H2-EB1: Histocompatibility 2, class II antigen E, beta 1
HFD: High-fat diet
iNOS: Inducible nitric oxide synthase
LPS: Lipopolysaccharide
LYL1: Helix-loop-helix transcription factor
LYVE1: Lymphatic vessel endothelial hyaluronan receptor 1
MCP-1: Monocyte chemoattractant protein-1
ME: Median eminence
MRC1: Mannose receptor C-type 1
MS4a7: Membrane spanning 4-domains A7
NO: Nitric oxide
P2ry12: Purinergic receptor P2Y12
PLBD1: Phospholipase B Domain Containing 1
PVN: Paraventricular nucleus of the hypothalamus
PVMs: Perivascular macrophages
ROS: Reactive oxygen species
SALL1: Spalt like transcription factor 1
scRNA-seq: Single-cell RNA sequencing
SLC2A5: Solute carrier family 2 member 5
SPIC: Spi-C Transcription Factor
VEGF: Vascular endothelial growth factor
